# Brain-derived neurotrophic factor as a possible predictor of electroconvulsive therapy outcome

**DOI:** 10.1038/s41398-019-0491-9

**Published:** 2019-05-24

**Authors:** Elisabeth Maria van Zutphen, Didi Rhebergen, Eric van Exel, Mardien Leoniek Oudega, Filip Bouckaert, Pascal Sienaert, Matthieu Vandenbulcke, Max Stek, Annemieke Dols

**Affiliations:** 1 Psychiatry, Amsterdam UMC, VU University Medical Center, Amsterdam Public Health Research Institute, Amsterdam, Netherlands; 2 GGZ inGeest Specialized Mental Health Care, Amsterdam, Netherlands; 3 Department of Epidemiology and Biostatistics, Amsterdam UMC, VU University Medical Center, Amsterdam Public Health, Amsterdam, Netherlands; 4 Psychiatry, Amsterdam UMC, VU University Medical Center, Amsterdam Neuroscience, Amsterdam, Netherlands; 5 Old-Age Psychiatry, University Psychiatric Center, KU Leuven, Leuven/Kortenberg, Belgium; 6 Academic Center for ECT and Neuromodulation (AcCENT), University Psychiatric Center, KU Leuven, Leuven/Kortenberg, Belgium

**Keywords:** Predictive markers, Molecular neuroscience, Depression, Prognostic markers

## Abstract

While brain-derived neurotrophic factor (BDNF) has been shown to predict response to pharmacotherapy in depression, studies in electroconvulsive therapy (ECT) are small and report conflicting results. This study assesses the association between pre-treatment BDNF levels and ECT outcome in severe late-life unipolar depression (LLD). The potential of BDNF as a clinical predictor of ECT outcome was subsequently evaluated. Characteristics associated with low and high BDNF subgroups were determined as well. Ninety-four patients diagnosed with LDD referred for ECT were included. Fasting serum BDNF levels were determined before ECT. Remission and response, measured with the Montgomery–Åsberg Depression Rating Scale, were the outcomes. The association between BDNF and ECT outcome was analysed with logistic regression and Cox regression. The clinical usefulness of BDNF was evaluated using the receiver operating characteristic (ROC) curve. Associations between clinical characteristics and low versus high BDNF levels were examined with *T* tests, chi-squared tests and Mann−Whitney tests. The odds of remission decreased with 33% for every five units increase of BDNF levels (OR 0.67, 95% confidence interval 0.47–0.96; *p* = 0.03); however, neither the association with time to remission nor the associations with response nor the adjusted models were significant. The area under the ROC (0.66) implied a poor accuracy of BDNF as a clinical test. Clinical characteristics associated with BDNF were inclusion site, physical comorbidities and duration of the index episode. To conclude, although there is an association between pre-treatment BDNF levels and ECT outcome, BDNF cannot be considered an eligible biomarker for ECT outcome in clinical practice.

## Introduction

Depression is the leading cause of disability worldwide^1^. To avoid long trajectories ending with treatment failure, predictors of treatment effect in depressed patients are needed. In older depressed patients, the presence of prior episodes, more severe symptoms, younger age of onset, comorbid dysthymia, more chronic diseases and less perceived social support were previously described to be associated with an unfavourable outcome^2–4^. Despite the fact that numerous features have been studied over the years, varying from clinical factors to genetic markers and neuroimaging techniques, powerful predictors of treatment effect are still lacking^5–7^.

Brain-derived neurotrophic factor (BDNF) is a protein that has received considerable attention in the field of depression. As postulated in the neurotrophin hypothesis, BDNF might be involved in the pathophysiology of depression^8^. Stress could induce a change in neurotrophic factors such as BDNF, which has a negative effect on neuronal plasticity^9^. Neuronal cell loss and atrophy, especially in the hippocampi, have been linked to depression and treatment outcome^10–12^. By diminishing neuronal plasticity, a change in factors as BDNF levels might thus impel depression. In line with this, it has been suggested that the effectiveness of antidepressant treatment could be explained by a normalization of neurotrophic factors and, subsequently, neuronal plasticity^9^.

Evidence supporting the role of BDNF in depression has been summarized in several meta-analyses. Indeed, peripheral BDNF levels were lower in acute MDD patients as compared to healthy controls^9,13,14^. Furthermore, a decrease in BDNF levels has been associated with the onset of depression^13^. In addition to this, an increase in peripheral BDNF levels following treatment with antidepressants has been associated with clinical improvement^9,14,15^. Altogether, even though the effects are small, these findings seem to support the neurotrophin hypothesis and it has been suggested that BNDF might be a biomarker for treatment with antidepressants^9,14^.

Electroconvulsive therapy (ECT) is the most powerful treatment for depression currently available, yet approximately one in three patients does not show a response^16,17^. Although it has been proven to be safe, it is intensive and may have unpleasant side effects^17,18^. Targeted therapeutic approaches are thus needed for ECT. Similar to antidepressant therapy, BDNF might be a treatment biomarker for ECT outcome. Meta-analyses showed that older age, pharmacotherapy resistance, shorter duration of the index episode and presence of psychotic symptoms were associated with favourable ECT outcome; nonetheless, the evidence on the potential association between baseline BDNF levels and ECT outcome was inconclusive^19–21^. A recent meta-analysis on BDNF and ECT showed that an increase in BDNF following ECT was not related to clinical outcome^22^, which differs from similar studies on antidepressants^9,14^. In ECT, a change in BDNF levels might thus not be related to the effectivity of the treatment, but perhaps baseline BDNF levels are associated with effectivity of the treatment. This has been established previously for antidepressant drugs^23,24^. In their meta-analysis, however, Polyakova et al.^22^ did not address the association of baseline BDNF levels and ECT outcome. To date, only two out of 11 studies on BDNF and ECT reported a significant difference in baseline BDNF levels between people who showed a response to ECT treatment and those who did not (for an overview, see Table 1). Small sample sizes ranging from seven to 61 and methodological differences such as mixed age groups and heterogenic psychiatric diagnoses might have resulted in inconsistent findings. Furthermore, BDNF levels have been linked with psychotic depression, age at onset and anxiety^25,26^. This suggests that BDNF levels may thus be relevant in only a specific subset of depressed people, which could also explain the inconsistent findings.

**Table 1:** Overview of current literature on baseline BDNF and ECT outcome

Paper	N, gender, age^a^ (years)	Diagnosis and severity of depression^a^	Blood sampling and overall BDNF levels	ECT characteristics	Psychotropic medication	Outcome measure	Main result (BDNF levels and *p*-value)
Bocchio-Chiavetto et al.^50^^b,c^	*N* = 23 ♀ 69.6% 53.0 (±17.4)	TR MDD (100% UP) 56.5% PS MADRS: 34.21 (±7.91)	In morning after overnight fast Storage −80 °C ELISA quantikine kit RD system Serum BDNF^a^ 27.6 (±9.1) ng/ml	3×/week, BL BP No. of sessions^a^: 7 (±2.0)	Continued, except in one participant	Remission (<9 on MADRS at two consecutive assessments), remission rate: 33.3%	Remitters: 27.1 ± 9.3 Non-remitters: 31.2 ± 8.4 *p* = 0.44
Marano et al.^55^^b,c^	*N* = 15 ♀26.7 % 50^d^	MDD (66.7% UP, 33.3 BiP) 40.0% PS HRSD-21^d^: 30.0 (±9.0)	Before ECT, Storage −80 °C ChemiKine BDNF sandwich ELISA kit Plasm BDNF^d^ 84.9 (±4.65) pg/ml	3×/week, BL or BF, BP No. of sessions^d^: 7	Continued	Response (−50% on HRSD-21), response rate: 86.7%	Responders: 83.1 ± 63.0 Non-responders: 119.5 ± 33.1 *p* = ND
Okamoto et al.^52^^b^	*N* = 18 ♀ 50% 60.6 (±14.1)	MDD or BiP, 27.7% PS HRSD-17: 23.1 (±4.5)	7.00 AM, after overnight fast Storage −80 °C BDNF Emax Immunoassay Kit Serum BDNF^a^ 11.0 (±11.2) ng/ml	3×/week, BL BP No. of sessions: 12	Continued	Response (−50% on HRSD-17), response rate: 66.7% Remission (<7 on HRSD-17), remission rate: 33.3%	Responders: 7.9 ± 9.9 Non-responders: 11.5 ± 11.0 *p* = ND, but it was reported to be not significant.
Fernandes et al.^60^^b^	*N* = 15 ♀ 66.7% 52.7 (±15.9)	TR MDD (73.3% UP, 26.7% BiP), 53.9% PS HRSD: 24.15 (±6.32)	1 day before ECT Storage −80 °C Sandwich ELISA, total protein measured by Lowry’s method Serum BDNF^d^ 0.3 (IQR 0.1) pg	3×/week, UL BP No. of sessions^a^: 11.2 (±1.4)	Continued	Response (−50% on HRSD), response rate: 73.3% Remission (<7 on HRSD), remission rate: 33.3%	BDNF levels and *p* value: ND No differences between ECT outcome groups.
Piccinni et al.^59^^b^	*N* = 18 ♀ 50% 44.9 (±17.0)	MDD (11% UP, 89%vBP), 44% PS HRSD-21: 26.4 (±6.0)	In morning after overnight fast Storage −20 °C ELISA Plasm BDNF^b^ 2.1 (±1.2) ng/ml	2×/week, BL BP No. of sessions^a^: 8.3 (±1.2)	Continued, except mood stabilizers	Remission (<10 on HRSD-21 at two consecutive assessments), remission rate: 44.4%	Remitters: 2.9 ± 1.3 Non-remitters: 1.5 ± 0.5 *p* = 0.02
Hu et al.^54^^c^	*N* = 28 ♀ 82.1% 41.0 (±14.8)	MDD HRSD-17: 31.39 (±4.65)	7:30 and 8:30 AM, 1 day before ECT Storage −20 °C Promega Enzyme-linked immunosorbent assay kit Serum BDNF^a^: 5.66 (±2.07) ng/ml	3×/week, BL	Discontinued	Response (−50% on HRSD-17), response rate: 85.7%	Responders:5.5 ± 1.9 Non-responders: 6.5 ± 3.4 *p* = ND
Gedge et al.^58^^c^	*N* = 11 ♀ 63.6% 46.5	MDD HRSD-17: 23.73 (±1.43)	7 days before ECT Storage −20 °C Sandwich ELISA Emax Immuno-AssaySystem Serum BDNF^a^: 9.95 (±1.94) ng/ml	3×/week Administered as per hospital protocol No. of sessions: 12	Continued	Response (−50% on HRSD-17), response rate: 45.5%	Responders:13.3 ± 6.7 Non-responders: 7.2 ± 5.2 *p* = ND
Stelzhammer et al.^56^^c^	*N* = 7 ♀ 71.4% 52.9 (±8.8)	TR MDD HRSD: 26.9 (±6.9)	Immediately before first ECT session Storage −80 °C Serum BDNF^a^: ND ng/ml	3×/week, BP Administered as per hospital protocol No. of sessions: 12	Discontinued, start with AD after 6 ECT sessions	Response (−50% on HRSD), response rate: 42.9%	Responders:20.4 ± 13.5 Non-responders: 22.7 ± 7.0 *p* = ND
Kleimann et al.^51b,c^	*N* = 11 ♀ 45.5% 47 (±16.5)	TR MDD, 73% PS MADRS: 34 (±8.3)	8–10 AM after overnight fast Storage −80 °C DuoSet enzyme-linked immunesorbent assay Development System Serum BDNF: ND pg/ml	3×/week, during 3.5 weeks Technique: as common practice in the facility	Continued	Response (−50% on MADRS), response rate: 54.5% Remission (<13 on MADRS), remission rate: 36.4%	Responders: 541.2 ± 294.9 Non-responders: 721.8 ± 364.1 *p* = 0.37
Freire et al.^57^^b^	*N* = 21 ♀ 90.5% 48.75 (±15.4)	DD (52.4% UP, 42.9% BiP), 47.6% PS HRSD-17: 26.04 (±6.62)	At admission Storage −80 °C ELISA, Milipore kit Serum BDNF: ND	3×/week, UL high dose, BP No. of sessions^a^: 8.8 (2.3)	Continued	Remission (<8 on HRSD-17), remission rate: 52.4%	BDNF levels: ND, but BDNF was higher in remitters than in non-remitters. *p* = 0.03
Ryan et al.^53^^e^	*N* = 61 ♀ 55.7% 52.7 (±15.4)	MDD (75.4% UP, 24.6% BiP), 24.6% PS HRSD-24: 31.2 (6.8)	7.30–9.30 AM, after overnight fast Storage −80 °C DuoSet® ELISA kit Plasm BDNF^a^: 1.09 (±0.19) log_10_ ng/ml	2×/week, 50.8% received moderate dose BL, 49.3% received high dose UL No. of sessions^a^: 8.2 (2.6)	Continued	Response (−60% on HRSD-24 and a final score of ≤16 on HRSD-24), response rate: 54.1% Remission (−60% on HRSD-24 and a score of ≤10 for 2 weeks on HRSD-24), remission rate: 41.0%	Responders: 1.08 ± 0.2 Non-responders: 1.11 ± 0.2 *p* = 0.87 Remitters: 1.07 ± 0.2 Non-remitters: 1.11 ± 0.2 *p* = 0.1
Van Zutphen, 2019^b^	*N* = 94 ♀ 68.1% 73.3 (±8.1)	MDD, 51.1% PS MADRS^d^: 34.0 (±12.0)	7.30 and 9.30 AM after overnight fast Storage −85 °C Emax Immuno Assay System Serum BDNF^a^: 18.1 (±6.6) ng/ml	2×/week, UL or BL BP No. of sessions^d^: 11 (6.3)	Continued in 38.3%	Response (−50% on MADRS), response rate: 81.9% Remission (<10 on MADRS), remission rate: 69.1%	Responders: 17.6 ± 6.7 Non-responders: 20.6 ± 5.6 *p* = 0.08 Remitters: 17.1 ± 6.8 Non-remitters: 20.4 ± 5.6 *p* = 0.02

*DD* depressive disorder, *MDD* major depressive disorder, *UP* unipolar depression, *BiP* bipolar depression, *PS* presence of psychotic features, *TR* treatment resistant, *HRSD* Hamilton Rating Scale for Depression, *MADRS* Montgomery–Åsberg Depression Rating Scale, *UL* unilateral, *BL* bilateral, *BF* bifrontal, *BP* brief pulse, *ND* not described

^a^Mean (SD)

^b^Prospective cohort study

^c^The BDNF levels stratified for ECT outcome of these studies were retrieved from the meta-analysis of Polyakova et al.^22^

^d^Median (IQR)

^e^Randomized controlled trial

In summary, low BDNF levels seem to relate to depression and pre-treatment BDNF levels could be associated with ECT outcome. The latter, however, is still unclear as previous studies were small, heterogenic and report contradictory results. Therefore, the first aim of this study is to clarify the association of BDNF and ECT outcome in a relatively large study population consisting of admitted unipolar depressed older patients eligible for ECT. We hypothesize that patients with lower BDNF levels will be more likely to benefit from ECT, since the majority of the previous studies (seven out of 11) reported lower BDNF levels in those with a favourable treatment outcome (Table 1). As BDNF appears to be a key molecule in neuronal plasticity and neuronal atrophy in the hippocampus has been linked to treatment outcome in depression, our findings will be reviewed in the context of hippocampal atrophy^10,27^. A second aim of this study is to assess the clinical relevance of the potential association between pre-ECT BDNF levels and ECT. By establishing the discriminative ability of BDNF, we hope to determine if pre-ECT BDNF levels could potentially guide clinicians in selecting eligible patients for ECT. A third aim of this paper is to examine the association of low and high BDNF levels with a wide variety of features, including socio-demographics, physical health characteristics, clinical features and magnetic resonance imaging (MRI) characteristics, as BDNF levels have been linked to clinical features other than the effect of ECT.

## Materials and methods

### Study sample

Data for this study were obtained from the Mood Disorders in Elderly treated with Electroconvulsive Therapy (MODECT)^28^, a naturalistic study including 110 older patients receiving ECT treatment in the context of severe unipolar depression. Respondents were recruited from two tertiary psychiatric hospitals (GGZ inGeest, Amsterdam, the Netherlands and University Psychiatric Center, KU Leuven, Belgium) between 1 January 2011 and 31 December 2013. Patients aged 55 years and older and diagnosed with MDD according to Diagnostic and Statistical Manual of Mental Disorders, Fourth Edition, Text Revision (DSM-IV-TR) were included^29^. Diagnoses were made by a psychiatrist and were validated with the Mini International Neuropsychiatric Interview (MINI)^30^. Patients with a DSM-IV-TR diagnosis of bipolar disorder or schizoaffective disorder were excluded, as well as patients with a major neurologic illness (e.g. dementia, Parkinson’s disease, stroke). Nurses trained in research conducted all questionnaires. The Ethical Review Board of the VU University Medical Center approved the study protocol of MODECT, as well as the Ethical Review Board of the Leuven University Hospitals. The study was conducted according to the Declaration of Helsinki (clinicaltrials.gov; NCT02667353). All patients gave written informed consent. The sample size for the MODECT study was calculated for our initial hypothesis (in 2009) of an ECT-induced change in hippocampal volume due to raised BDNF levels. In the current study, all respondents with missing values on BDNF or depression severity prior to and post ECT were excluded from analyses (*n* = 16). For the current study we thus used all samples available, and this is the largest sample size on this topic of pre-ECT BDNF levels and ECT outcome to date. Attrition analyses showed that the excluded patients did not differ from the studied population in terms of socio-demographics and baseline severity of depression (data not shown).

### Measurements

#### ECT outcome

Severity of depression was assessed with the Montgomery Åsberg Depression Scale (MADRS) before, during (weekly) and after ECT course^31^. Response was defined as a decrease of ≥50% of MADRS score, as compared to baseline MADRS score. Remission was defined as a MADRS score <10 points at two consecutive assessments^32^.

#### BDNF

In the week before the start of ECT, blood samples were collected between 7.30 and 9.30 A.M. after an overnight fast. Emax Immuno Assay System from Promega (Madison, WI, USA), catalogue number G7610, was used to determine the BDNF protein levels in ng/ml in serum. The procedure has been described in more detail previously by Bouckaert et al.^33^. To increase contrast, BDNF levels were rescaled in units of five. Storage time in days was included in the analysis as well^34^.

#### Covariates

A variety of variables, including socio-demographics, physical health and lifestyle characteristics, clinical characteristics, MRI characteristics, and ECT characteristics, were examined. Socio-demographics included: age, gender, inclusion site (Amsterdam or Leuven), marital status (never married/married/divorced/ widowed) and level of education. Level of education was categorized as low (no education, primary school), intermediate (high school, vocational training) or high (college, university). Physical health and lifestyle characteristics included current smoking status (yes/no), current alcohol use (yes/no) and presence of chronic physical comorbidities (yes/no), and were assessed during a semi-structured interview. Clinical factors included presence of psychotic features (yes/no) as measured with the MINI^30^ and verified by clinical judgement at baseline, age at onset of first depression (early/late, cut-off age at onset: 55 years), duration of the current episode in months, antidepressant resistance score (evaluated with Antidepressant Treatment History Form (ATHF)^35^), cognitive functioning prior to ECT (evaluated with Mini Mental State Examination (MMSE)^36^) and MADRS scores prior to and post ECT. MRI characteristics included scores on Global Cortical Atrophy scale (GCA)^37^ and Scheltens’ scale for Medial Temporal Atrophy (MTA), which is a measure for hippocampal atrophy^38^. Whole-brain MRI scans were made at baseline. In Amsterdam, General Electric Signa HDxt (Milwaukee, WI, USA) was used and in Leuven, Philips Intera (Best, the Netherlands) was used. For a more detailed description of MRI techniques and measuring instruments, we refer to Dols et al.^28^. All images have been reviewed by an experienced neuro-radiologist, unaware of patient-related clinical information. Finally, ECT characteristics included number of ECT sessions and duration of ECT treatment in days.

#### ECT procedure

Patients received ECT according to the prevailing Dutch standards^39^. The Thymatron System IV (Somatics, LLC, Lake Bluff, IL, USA) (maximum energy 200%, 1.008 C) was used to give all treatments. Stimulus intensity was based on empirical dose titration at the first session, which is the initial seizure threshold times six for right unilateral ECT, and initial seizure threshold times 1.5 for bilateral ECT. All patients received brief-pulse ECT (0.5–1.0 ms). Psychotropic medication was ceased from at least 1 week before the start of ECT treatment or, if cessation was considered impossible, remained unchanged from 6 weeks before the start of the ECT treatment until treatment was finished. After the start of ECT, patients were evaluated weekly to determine the clinical condition. If no improvement was seen by the treating psychiatrist after six unilateral sessions or if the clinical conditions worsened (e.g. increase of MADRS scores, suicidality, harmful psychotic features, dehydration, weight loss), a change to bilateral treatment was considered necessary. ECT was discontinued when the MADRS score <10 points at two consecutive ratings with a weekly interval or when no additional improvement was seen during the last two ECT sessions after at least six unilateral and six bilateral sessions. The number of ECT sessions ranged between four and 29 (median: 11 IQR 6.25). Three patients started with bilateral ECT. Out of the 91 patients that started with unilateral ECT, 30 patients switched to bilateral ECT after a median of seven unilateral treatments. More details can be found in Dols et al.^28^.

### Statistical analyses

IBM SPSS statistics 23 was used for all data analyses. To examine group differences, *χ*^2^ tests (exact methods) were used to analyse all categorical variables and independent *t* tests were used for all continuous variables with a normal distribution. Variances of the groups were compared with Levene’s Test for Equality of Variances and were not significantly different. Non-normally distributed continuous variables were transformed using the natural logarithm, or, if the former did not help, Mann−Whitney *U* tests were used to compare groups.

Logistic regression analysis was used to determine the association between the BDNF levels and ECT outcome. Next, the time to event (remission or response) was analysed, with duration of treatment in days as time indicator. Differences in duration of treatment between the two BDNF subgroups were analysed with independent *t* tests (original data or transformed data using the natural logarithm) or Mann−Whitney tests, depending on normality. Logrank tests were used to compare time to event distributions and, with Cox proportional hazards model, the effect of BDNF adjusted for putative confounders on time to event was assessed. The assumption of proportionality of hazard was checked and confirmed by tests of the interaction of time with the covariate. All analyses were adjusted for factors potentially influencing BDNF blood levels, that were selected based on a significant difference between BDNF subgroups as well as a theoretical framework. Because of small proportions of non-remission and non-response, respectively, we were unable to test one fully adjusted model^40^. For this reason, we tested three adjusted models separately: one adjusted for socio-demographics (age, gender, educational level), one adjusted for potential differences in execution of the protocol between sites and a known confounder of BDNF levels (inclusion site and storage time) and one model adjusted for clinical features based on the significant differences between low and high BDNF subgroups (Table 2) (duration of index episode and presence of physical comorbidities)^34^. Correlation, and hence putative multicollinearity in regression analyses, of covariates was examined by Spearman’s rank-order correlation coefficient, with a correlation coefficient ≥0.50 as an indicator of multicollinearity. Since inclusion, and thus treatment and blood collection, found place at two different sites, an interaction between inclusion site and BDNF was examined in the crude models. Earlier reports described an interaction between BDNF and gender and age, respectively; hence BDNF × gender and BDNF × age interactions were examined in the crude models^41^. Analyses were stratified if interaction terms were statistically significant. The results of all regression analyses are presented as the effects for a difference of five units BDNF, instead of one unit BDNF, as this facilitates interpretation of the effects^42^. Diagnostic performance of BDNF as a measure for treatment effect was determined using receiver operating characteristic (ROC) curves. Putative cut-off values for BDNF to calculate sensitivity and specificity were based on the 20th, 30th, 40th, 50th, 60th 70th and 80th percentiles. The Youden-index was used to determine the best cut-off value^43^. The BDNF value corresponding to the highest Youden-index was used to dichotomize BDNF in low and high BDNF subgroups, in order to examine the association of BDNF and clinical characteristics. All tests were two-tailed and statistical significance was set as *p* < 0.05. Interaction terms were considered significant at *p* < 0.10. We studied two outcome measures (remission and response) in two types of regression methods (logistic regression and Cox regression). The subsequent confounder-adjusted models should not be viewed as new tests, and therefore there were a total of four tests, not requiring correction for multiple testing.

**Table 2:** Main characteristics

	Total	Low BDNF^a^	High BDNF	*χ*²/*t*/*U* (df) *p* value
	94 (100)	*N* = 47 (50%)	*N* = 47 (50%)	
Socio-demographics				
Age, mean (SD)	73.3 (8.1)	74.3 (7.7)	72.2 (8.4)	1.3 (92) 0.20
Gender, female	64.0 (68.1)	31.0 (66.0)	33.0 (70.2)	0.2 (1) 0.83
Inclusion site: Amsterdam	52.0 (55.3)	18.0 (38.3)	34.0 (72.3)	11.0 (1) < 0.01
Educational level				0.4 (2) 0.82
Low	13.0 (15.9)	8.0 (18.2)	5.0 (13.2)	
Intermediate	46.0 (56.1)	24.0 (54.5)	22.0 (57.9)	
High	23.0 (28.0)	12.0 (27.3)	11.0 (28.9)	
Physical health and lifestyle characteristics				
Smoking, currently *n* = 78	21.0 (26.9)	7.0 (20.0)	14.0 (32.6)	1.5 (1) 0.31
No alcohol use *n* = 88	30.0 (34.1)	27.0 (60.0)	31.0 (72.1)	1.4 (1) 0.27
Physical comorbidities present	77.0 (81.9)	33.0 (70.2)	44.0 (93.6)	8.7 (1) 0.01
Clinical characteristics				
MDD with psychotic features	48.0 (51.1)	27.0 (57.4)	21.0 (44.7)	1.5 (1) 0.30
Late onset (>55 year) of depression	54.0 (57.4)	29.0 (61.7)	25.0 (53.2)	0.7 (1) 0.53
Duration of index episode in months, median (IQR) *n* = 88	6.0 (10.0)	5.0 (5.8)	7.5 (11.3)	−2.1 (86) 0.04^b^
ATHF resistance score, median (IQR), *n* = 91	4.0 (5.0)	4.0 (5.0)	5.0 (5.0)	−1.3 (89) 0.20^b^
MMSE score before ECT-treatment, median (IQR), *n* = 84	25.5 (6.0)	25.0 (6.0)	26.0 (6.0)	874.5; 0.95
MADRS-scores, median (IQR)				
Before ECT-treatment	34.0 (12.0)	34.0 (10.0)	34.0 (15.0)	0.2 (92) 0.85
After ECT-treatment	6.0 (9.0)	6.0 (6.0)	7.0 (14.0)	−2.1 (92) 0.04^b^
Response to ECT treatment	77.0 (81.9)	43.0 (91.5)	34.0 (72.3)	5.8 (1) 0.03
Remission after ECT treatment	65.0 (69.1)	39.0 (83.0)	26.0 (55.3)	8.4 (1) 0.01
MRI characteristics				
MRI before ECT	77.0 (81.9)	39.0 (83.0)	38.0 (80.9)	0.1 (1) 1.00
GCA score, median (IQR)	1.0 (1.0)	1.0 (1.0)	1.0 (2.0)	629.0; 0.21
MTA score, median (IQR)	1.0 (1.5)	1.0 (1.0)	1.0 (1.1)	636.0; 0.27
ECT characteristics				
Duration of ECT treatment (days), median (IQR)	38.5 (24.0)	38.0 (21.0)	39.0 (24.0)	−0.5 (92) 0.64^b^
Continuation of psychotropic medication	36 (38.3)	15 (31.9)	21 (44.7)	1.6 (1) 0.29
BDNF characteristics, mean (SD)				
BDNF (ng/ml)				
Pre-ECT	18.1 (6.6)	12.9 (3.8)	23.3 (4.3)	−12.4 (92) < 0.01
Post-ECT	18.1 (6.2)	14.5 (5.4)	21.6 (4.8)	−6.3 (80) < 0.01
Storage time (days)	831.1 (294.6)	826.5 (297.8)	835.8 (294.5)	−0.2 (92) 0.88

Data shown as *n* (%) unless reported otherwise. In case of missing data, the number of complete cases (*n*=) for that variable is presented in the left column.

*BDNF* brain-derived neurotrophic factor, *IQR*interquartile range, *ng* nanograms, *ml* millilitres, *SD* standard deviation

^a^BDNF levels have been split on the 50th percentile (17.9 ng/ml), resulting in low and high BDNF subgroups

^b^Skewed data, T-tests performed on log-transformed data

## Results

### Demographic and clinical characteristics

In total, 94 subjects were included, with an age ranging from 55 to 92 years. The majority of participants were female (Table 2). The mean depression severity score at baseline was 33.6 (SD: ±9.0) out of 60 points and all participants were treated with at least one (range 1–5) antidepressant before applying for ECT. Psychotropic medication was continued during ECT in 38.3% of the respondents. The medication included antipsychotics (haloperidol, quetiapine, olanzapine), tricyclic antidepressants (imipramine, amitriptyline, nortriptyline, mirtazapine) and lithium.

### BDNF and recovery

Baseline BDNF levels ranged from 5.8 to 35.6 ng/ml, with a mean value of 18.1 ng/ml (SD: ±6.6). Mean BDNF levels differed significantly between remitters (17.1, SD: ±6.8) and non-remitters (20.4, SD: ±5.6) (*t* = 2.3, *p* = 0.02, 95% CI: 0.5–6.2); however, the blood levels in responders (17.6, SD: ±6.7) and non-responders (20.6, SD: ±5.6) did not (*t* = 1.8, *p* = 0.08, 95% CI: −0.4–6.6). Likewise, logistic regression showed a significant relation between BDNF and remission (OR: 0.67, 95% CI: 0.47–0.96, *p* = 0.03), but not between BDNF and response (OR: 0.70, 95% CI: 0.47–1.05, *p* = 0.08). The effects of BDNF are expressed in units of five. For example, the crude odds ratio (OR) of BDNF on remission can be interpreted as: an increase of five units in BDNF is associated with a 0.67 times lower odds on remission, or as: if two persons differ five units in BDNF, the odds on remission of the person with a higher BDNF level are 33% (1–0.67) lower than the odds for remission of the person with a lower BDNF level.

Three adjusted models were made, because adjusting for all putative confounders at once was not possible. The first adjusted model consisted of BDNF, age, gender and educational level. The OR was 0.59 (95% CI: 0.39–0.90, *p* = 0.01) for remission and 0.68 (95% CI: 0.43–1.09, *p* = 0.11) response. The second adjusted model consisted of BDNF, inclusion site and storage time in days. In this model, the OR of BDNF on remission was 0.72 (95% CI: 0.49–1.07, *p* = 0.10) and the OR for BDNF on response was 0.75 (95% CI: 0.48–1.18, *p* = 0.22). The third adjusted model included BDNF, duration of the index episode and presence of somatic disease. The OR was 0.71 for remission (95% CI: 0.49–1.03, *p* = 0.07) and 0.75 for response (95% CI: 0.49–1.15, *p* = 0.19).

### Speed of recovery

**Fig. 1:**
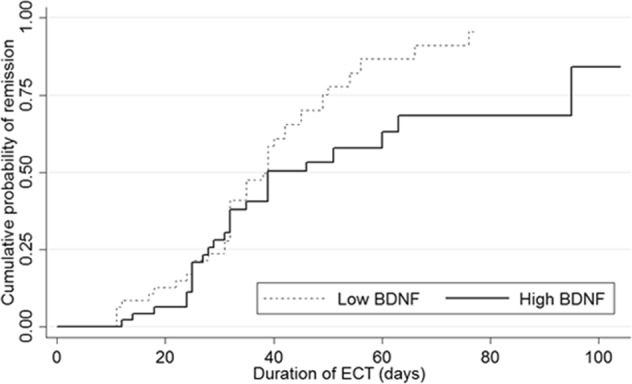
Kaplan−Meier curve for remission stratified by the BDNF subgroup based on the 50th percentile. Distributions were compared with the logrank test (*p* < 0.05)

The duration of ECT treatment ranged from 11 to 104 days, which did not differ between the BDNF subgroups (Table 2). Non-remitters received ECT for a longer period than remitters (*t* = 2.9 (92), *p* < 0.01). The same applied to non-responders and responders (*t* = 3.3 (92), *p* < 0.01). Log rank tests showed that time to remission distributions for low and high BDNF subgroups, based on 50th percentile, were statistically significantly different (*χ*^2^ = 4.0 (1), *p <* 0.05) (Fig. 1). The same did not apply to time to response (*χ*^2^ = 2.2 (1), *p* = 0.14). The hazard ratio (HR) of remission or response, including correction for factors potentially affecting BDNF levels, did not reach significance. The unadjusted analyses showed a HR of 0.87 (95% CI: 0.71–1.06, *p* = 0.16) on remission and a HR of 0.92 (95% CI: 0.77–1.11, *p* = 0.38) on response. The three adjusted models, one adjusted for socio-demographics, one adjusted for inclusion site and storage time in days and one adjusted for duration of the index episode and somatic diseases, showed HRs of 0.87 (95% CI: 0.70−1.07, *p* = 0.19), 0.90 (95% CI: 0.73–1.12, *p* = 0.36) and 0.90 (95% CI: 0.73–1.12, *p* = 0.34), respectively, for remission, and 0.95 (95% CI: 0.78–1.15, *p* = 0.56), 0.96 (95% CI: 0.79–1.17, *p* = 0.66) and 0.96 (95% CI: 0.79–1.16, *p* = 0.65), respectively, for response. Interaction terms for BDNF and, respectively, age, gender and inclusion site, were not significant in both Cox regression and logistic regression (data not shown).

### Diagnostic performance

The ROC curve used to evaluate the diagnostic performance of BDNF as a predictor of ECT outcome had an area under the curve of 0.66 (95% CI 0.55–0.77) for remission and 0.64 (95% CI 0.51–0.77) for response. Potential cut-off values based on percentiles with corresponding sensitivity, specificity and Youden-index are shown in Table 3.

**Table 3:** Potential cut-off values of BDNF with corresponding sensitivity and specificity

	Percentile	BDNF	Sensitivity	Specificity	Youden-index
Remission	20	11.8	0.2	0.9	0.2
	30	15.2	0.4	0.9	0.2
	40	16.5	0.5	0.8	0.3
	50	17.9	0.6	0.7	0.3
	60	19.8	0.7	0.6	0.2
	70	21.1	0.8	0.4	0.2
	80	23.6	0.8	0.2	0.1
Response	20	11.8	0.2	0.9	0.1
	30	15.2	0.3	0.9	0.2
	40	16.5	0.4	0.8	0.3
	50	17.9	0.6	0.8	0.3
	60	19.8	0.6	0.6	0.2
	70	21.1	0.7	0.4	0.1
	80	23.6	0.8	0.2	0.0

Youden index = sensitivity + specificity – 1

*BDNF* brain-derived neurotrophic factor level in ng/ml

### Characteristics associated with BDNF

The highest Youden-index was 0.3, which corresponds to a cut-off value of 17.9 ng/ml (Table 3). This value was used to split the study population in a high and a low BDNF subgroup. Table 2 shows the distribution of various characteristics across low and high BDNF subgroups. Low and high BDNF subgroups differed statistically significantly in terms of inclusion site, presence of chronic physical comorbidities and duration of the index episode.

## Discussion

In this study, baseline BDNF levels were lower among respondents with a favourable ECT outcome. Interestingly, BDNF had a statistically significant association with remission only and not with response nor the speed of remission and response (i.e. Cox regression analyses). The specificity and sensitivity of BDNF, however, were quite low, making BDNF not an eligible biomarker for clinical practice.

### BDNF and ECT outcome

The association between BDNF levels and ECT outcome has been examined in several other studies (Table 1). The results were inconsistent, possibly due to small sample sizes and differences in age, diagnosis, type of specimen, sampling method and (dis)continuation of psychotropic medication, which could all influence BDNF levels or treatment effect^15,44–49^. Four studies found non-significantly lower levels among patients with a favourable ECT outcome^50–53^. Three other studies also found lower levels among responders; however, the between-group differences were not tested^54–56^. On the other hand, three studies found higher levels to be associated with a favourable ECT outcome, of which two were statistically significant^57–59^. One study reported that the difference in BDNF levels was not significant; however, the actual BDNF levels were not described^60^.

We are thus the first to report a significant association between low BDNF levels and favourable ECT outcome and, with a sample size of 94, the current study is by far the largest study to date on this topic (Table 1). Previous studies thus might have been underpowered to detect a (significant) difference. In addition to this, with a range from 0.0003 ng to 47.3 ng/ml, BDNF levels differ greatly between studies (Table 1). The question arises if results based on such varying BDNF levels are comparable.

In the current study, low BDNF levels were associated with a favourable treatment outcome, but only between remitters and non-remitters. The mean BDNF levels of remitters and responders, however, were comparable (17.1, SD: ±6.8 and 17.6, SD: ±6.7), respectively), and the same applied to non-remitters and non-responders (20.4, SD: ±5.6 and 20.6, SD: ±5.6, respectively). The mean differences in BDNF levels between responders and non-responders (3.1, SD: ±1.8) and remitters and non-remitters (3.4, SD: ±1.4) were also approximately equal. With only 17 patients (18%) who showed no response, ECT was very effective in the current study sample. Considering the similarities in BDNF levels described above, the proportion of non-responders might have been too low to detect a *statistically significant* difference between responders and non-responders. The current results indicate that depressed older people with lower levels of BDNF are more likely to achieve remission with ECT, but that the speed of achieving remission is not associated with BDNF levels.

After correction for various factors potentially influencing BDNF levels, analyses became mainly insignificant. Although this could mean that the association between BDNF and ECT outcome was largely explained by these factors, it is also very likely that the adjusted analyses were underpowered. The high proportions of remission and response resulted in a small number of patients not experiencing treatment effect. This drastically limits the number of covariates that can be added to the model and an increase of the *p* value could be expected^40^. It should be noted though that the (rounded) point estimates (OR and HR) and 95% confidence intervals hardly change after adding confounders to the analysis model.

### BDNF, ECT outcome and the hippocampus

The association between lower BDNF levels and favourable ECT outcome might be related to hippocampal volume. Smaller hippocampal volume has been linked to a favourable ECT outcome^61,62^ and particular hippocampal subfield volumes have been described to predict response to ECT^63^. As a neurotrophic factor, BDNF levels could be associated to features such as hippocampal size. Although the MTA scores did not differ between low and high BDNF subgroups in the current study, other studies have shown that hippocampal volumes were positively correlated with BDNF levels^33,45^. This could imply that smaller hippocampal volume, and not necessarily atrophy, is associated with BDNF levels. Collectively, these studies outline a potential mediating effect of hippocampal volume on the association between lower BDNF levels and favourable ECT outcome.

### Clinical features and BDNF

Low BDNF levels were significantly associated with inclusion site Leuven, lack of physical comorbidities and shorter duration of the index episode (Table 2). BDNF levels have been related to a variety of somatic diseases, including inflammatory diseases, diabetes mellitus, cardiovascular diseases and asthma^34,64^. Only shorter duration of the index episode was previously reported to be associated with favourable treatment outcome of ECT as well^19^. The association of shorter duration of the index episode with both low BDNF levels and favourable treatment outcome is in line with our finding of lower BDNF levels increasing the odds of remission. In contrast to our findings concerning duration of the index episode, we did not find an association between BDNF levels and other previously reported predictors of a favourable ECT outcome^19,21^. Bus et al. reported that BDNF levels were associated with age in women only^34^. As the mean age of women across low and high BDNF subgroups did not differ significantly (*p* = 0.46), we were unable to confirm the findings of Bus et al.^34^. The study population of Bus et al.^34^ had a mean age of 61.2 years, ranging from 50 to 72 years. The mean age in the current study was 73.3 years and ranged from 55 to 92 years. So, not only was the mean age 12 years higher in the current study, it was also higher than the oldest person included in the study of Bus et al.^34^, suggesting that the difference might be present only in younger-old subgroups. Another explanation, as suggested by Bus et al.^34^, could be that the current study is underpowered to detect an age difference. The presence of psychotic symptoms did not differ among the BDNF subgroups. This confirmed the results of a previous study^65^; however, a weak correlation between BDNF and thought disturbance has been described as well^25^.

### Discriminative ability and diagnostic potential of BDNF

Although the odds of remission decreased with an increase of BDNF levels, the performance of BDNF as a biomarker for ECT remission in our study sample was quite low^66^. This questions the potential of BDNF as a clinical tool to select patients eligible for ECT.

### Strengths and limitations

Our findings should be interpreted in the context of the following strengths and limitations. A strength of the current study is the relatively large sample size. In the existing literature, most study sizes range from 11 to 23 participants, with the largest to date being *N* = 61 (Table 1). We believe that psychotropic medication had limited influence on the BDNF levels in the current study^15,48^. The majority of patients (*N* = 58; 61.7%) discontinued their psychotropic medication at least 1 week before ECT and the patients who continued their medication were equally distributed over BDNF (Table 2), remission and response subgroups (data not shown). In addition to this, the studied population was homogenous in respect to psychiatric diagnosis (only unipolar depression, inpatients sample), as evidence on the comparison of BDNF levels between various psychiatric diseases remains contradicting^46,67^.

In contrast to these strengths, limitations should be mentioned. As in many other studies, BDNF levels were measured in peripheral blood. Whereas BDNF can cross the blood−brain barrier, the exact source of peripheral BDNF remains unclear^68–70^. Furthermore, although correlations between peripheral and central BDNF levels have been reported in mammals, how BDNF levels in serum are related to levels of BDNF within the brain in humans remains speculative^71^. Another limitation of the current study was the small number of non-remitters and non-responders, which hampered the adjusted analyses. The results of our adjusted analyses should thus be interpreted with caution. The study design could be criticized too. For example, we did not have a control group, which limits the interpretation of the between-group differences as well as it hampers comparison with other studies. Also, the multicentre design complicates quality assurance. Place of inclusion was no significant effect modifier; nevertheless, mean BDNF levels differed significantly between the two places (mean difference: 5.8, *p* < 0.001). This might be because of differences in blood sampling methods. On the other hand, as previously described by Dols et al.^28^, the two subpopulations from Amsterdam and Leuven are not identical: e.g. marital status, physical comorbidities and periventricular white matter hyperintensities differed among these subpopulations. In addition to this difference in the presence of physical comorbidities across inclusion places, BDNF levels in the current study were associated with the presence of physical comorbidities too. For the association of BDNF and ECT outcome in the two subpopulations from Amsterdam and Leuven, stratified analyses showed effect sizes that were somewhat smaller yet comparable to those in the whole sample, but statistical significance was lacking (data not shown). Finally, despite the large array of putative confounders, data on other putative factors that were previously reported to affect BDNF levels, e.g. BMI and physical exercise, were lacking^34^. Post hoc analyses showed that seasonality did not affect the associations between BDNF and ECT outcome (data not shown)^72^.

### Conclusion

In conclusion, low BDNF levels were associated with better physical health, early referral for ECT and remission after ECT. The potential of BDNF as a clinical test for ECT outcome, however, could not be confirmed in the current study. Although these findings do not support strong recommendations to further study the predictive value of BDNF in ECT, conducting a meta-analysis should be a priority over establishing a new individual study.
